# Reply to: Disability Milestones and Death in Parkinson's Disease under Subthalamic Neurostimulation

**DOI:** 10.1002/mdc3.13840

**Published:** 2023-07-31

**Authors:** Nils Schnalke, Björn Falkenburger

**Affiliations:** ^1^ Department of Neurology University Hospital Carl Gustav Carus Dresden Dresden Germany; ^2^ Center for Neurodegenerative Diseases within the Helmholtz Association (DZNE) Dresden Germany

**Keywords:** deep brain stimulation (DBS), nucleus subthalamicus (STN), morbidity milestones, neurofilament, neurodegeneration

We thank Mahlknecht and colleagues for their thoughtful comments on our recently published research in which we examined the occurrence of disability milestones (frequent falls, hallucinations, dementia, and nursing home placement) and their relationship to death in a cohort of 162 patients with Parkinson's disease (PD) and deep brain stimulation of the subthalamic nucleus (STN‐DBS).^1^

In response to our work, they reanalyzed data from one of their previous studies comparing patients with deep brain stimulation (DBS) to a matched control group.^2^ Similar to our observations, they found that psychosis was the only milestone to occur less frequently in patients with STN‐DBS, which could be a consequence of the lower daily dose of dopaminergic medication. All other milestones showed comparable incidences in patients with STN‐DBS and in the matched control group without DBS. As in our study, the time from milestone occurrence to death was the same in both groups.

Mahlknecht and colleagues argue that their observations could be taken as a hint that STN‐DBS modifies the course of PD, whereas our interpretation has been more conservative. We agree with the notion that we cannot expect data from randomized controlled trials about the long‐term effects of STN‐DBS and that we have to base our counseling on retrospective analyses like the ones cited. We definitely agree that STN‐DBS is a powerful symptomatic treatment that significantly and continually improves patients’ quality of life.

We draw the reader's attention to a second notion that is embedded in these studies. The frequency of disability milestones and the time between milestone occurrence and death is similar in these two independent cohorts of patients with STN‐DBS, in the cohort of patients without STN‐DBS, and in the original cohort by Kempster and colleagues.^3^

Taken together, this evidence suggests that the final stage of PD is much more homogenous than earlier stages of PD, raising the question what drives disease progression during this final stage of PD. Rödström and colleagues^4^ recently demonstrated accelerated neurodegeneration in late‐stage PD as reported by serum neurofilament. We also measured serum neurofilament in our cohort, finding significantly higher serum neurofilament in patients with clinical milestones.^5^ Given that serum neurofilament reports the rate of neurodegeneration, these findings suggest that the functional decline in late stage of PD is driven by accelerated neurodegeneration, which could be explained by the exponential spread of Lewy pathology across the nervous system.

For counseling patients, it should be noted that these findings apply to patient cohorts, and because of the significant differences between individual patients, the time between milestone onset and death cannot be predicted for an individual patient. For clinical trials investigating disease modifying effects, however, onset of disability milestones could be used as a robust and relevant outcome measure.

## Author Roles

(1) Manuscript Preparation: A. Writing of the First Draft, B. Review and Critique.

N.S.: 1A, 1B.

B.F.: 1B.

## Disclosures

**Ethical Compliance Statement**: There was no further informed patient consent necessary for this work. We confirm that we have read the Journal's position on issues involved in ethical publication and affirm that this work is consistent with those guidelines.

**Funding Sources and Conflicts of Interest**: There is no funding to report and the authors have no conflicts of interest to disclose.

**Financial Disclosures for the Previous 12 Months**: N.S. reports no external funding related to the conduct of this study. Outside of the submitted work, he has received travel grants by Abbott, EverPharma, and the International Parkinson's Foundation. B.F. reports no external funding related to the conduct of this study. Outside of the submitted work, he reports grants from the German Research Foundation (DFG) and speaker honoraria from AbbVie, Stadapharm, Desitin, Zambon, and Bial.
